# Creating Protected Areas on Public Lands: Is There Room for Additional Conservation?

**DOI:** 10.1371/journal.pone.0148094

**Published:** 2016-02-05

**Authors:** Rodrigo A. Arriagada, Cristian M. Echeverria, Danisa E. Moya

**Affiliations:** 1 Millennium Nucleus Center for the Socioeconomic Impact of Environmental Policies (CESIEP), Santiago, Chile; 2 Interdisciplinary Center for Intercultural and Indigenous Studies (ICIIS), Center for Applied Ecology and Sustainability (CAPES), Department of Ecosystems and Environment, Pontificia Universidad Católica de Chile, Santiago, Chile; 3 Department of Forest Management and Environment, Universidad de Concepción, Concepción, Chile; National University of Singapore, SINGAPORE

## Abstract

Most evaluations of the effectiveness of PAs have relied on indirect estimates based on comparisons between protected and unprotected areas. Such methods can be biased when protection is not randomly assigned. We add to the growing literature on the impact of PAs by answering the following research questions: What is the impact of Chilean PAs on deforestation which occurred between 1986 and 2011? How do estimates of the impact of PAs vary when using only public land as control units? We show that the characteristics of the areas in which protected and unprotected lands are located differ significantly. To satisfactorily estimate the effects of PAs, we use matching methods to define adequate control groups, but not as in previous research. We construct control groups using separately non-protected private areas and non-protected public lands. We find that PAs avoid deforestation when using unprotected private lands as valid controls, however results show no impact when the control group is based only on unprotected public land. Different land management regimes, and higher levels of enforcement inside public lands may reduce the opportunity to add additional conservation benefits when the national systems for PAs are based on the protection of previously unprotected public lands. Given that not all PAs are established to avoid deforestation, results also admit the potential for future studies to include other outcomes including forest degradation (not just deforestation), biodiversity, wildlife, primary forests (not forests in general), among others.

## Introduction

Changes in land use are among the most important human alterations of the Earth´s land surface [1,2]. As a response, private actors and governments have significantly increased the number of protected areas (PAs) to conserve natural landscapes [3,4]. In the context of less developed countries, not only has the public demand for land for conservation increased, as revealed by government policies, but so has the overall demand for land for agricultural production, urban and suburban expansion, and other activities [5]. Despite the expansion of PAs in less developed countries, including Chile, not much is known both about the policy process determining the establishment of PAs, and about how successful such areas have been in contributing to their conservation objectives [6]. To inform decision makers regarding any new investments in land conservation, a better understanding is needed regarding where previous PAs have been placed and how effective these past conservation investments have been.

In most countries, governments have not randomly distributed PAs geographically [4], in part due to historical patterns of state land ownership. In situ biodiversity conservation, for example, has traditionally relied on PAs, and historically such areas often consisted of already public lands [7]. Regardless of the scientific and public choice factors underlying the establishment of PAs, if their resulting distribution is biased to favor areas of lower conservation threats, then most previously used methods will tend to overestimate the effectiveness of protection for reducing harmful land use and land cover changes (LULCC) [8,9]. The present paper addresses these selection effects.

In terms of effectiveness of PAs, the issue of the impact of PAs on LULCC is complicated because avoided changes to land are not directly measurable. Daniela Miteva et al. [6] summarize the studies that use rigorous empirical designs to quantify the impacts of PAs, finding that these studies have focused predominantly on the effectiveness of PAs in preventing deforestation [6]. Previous studies have suggested that the impact of PAs can mainly be explained by threats of land conversion and enforcement [10–12]. Most of these studies apply empirical designs whereby PAs are compared to observationally similar unprotected lands, but without distinguishing between private and government lands as comparable control areas. Therefore, this empirical strategy ignores that traditional PA systems are usually based on converted, long-held government lands, which perhaps have different land use regimes and different levels of enforcement relative to private lands. Within this context, this research addresses an important question for the analysis of the impact of PAs—what are the effects of establishing PAs in comparison with the counterfactual scenario obtained from unprotected public lands?

This paper corrects the problems with selection bias and the proper choice of controls in evaluating the impact of PAs by presenting causal estimates of their effectiveness in Chile. The paper demonstrates that evaluations can be substantially improved by controlling for biases along dimensions that are observable. We address two main research questions: What is the impact of Chilean PAs on avoided deforestation which occurred between 1986 and 2011? And, how do estimates of the impacts of PAs vary when using only public land as control units? This research effort constitutes the first impact evaluation of one of the oldest PA systems in Latin America, which protects one of the few remaining extensive temperate rainforests in the world. In terms of methodology, to adequately estimate the effects of PAs, we employed matching methods to define adequate control groups, but not as has been done in previous research, although we recognize previous attempts to stratify the sample in the evaluation of the impact of PAs (e.g. [13])

## PAs, LULCC and the Chilean System of Pas

PAs are, and will remain, the cornerstone of global conservation efforts [14]. An increasing human population and standard of living, and demand for multiple ecosystem services, will intensify competition for land inside and outside PAs. In the context of PAs and avoided deforestation, protection effectiveness will depend on the conservation opportunity costs of keeping land in forest. [5] and [10], presents a framework for considering PAs’ impacts on LULCC where protection effectiveness depend on rents determined by opportunity costs. Therefore, if we consider PAs impact on avoided deforestation, PAs may remain forested due to the protection itself (i.e. *de jure* protection) or because the landscape characteristics of the protected lands discourage LULCC (i.e. *de facto* protection). In the latter case, protection may have no impact at all. Accordingly, the primary question PAs administrators should ask is whether the conservation scheme has a sufficiently large “additionality” which is the difference in conservation between the with-PAs scenario and the without-PAs baseline [15]. At the end, the impact of PAs is an empirical question that requires rigorous empirical evidence to be answered. This is the research challenge that this paper will address in the context of one of the oldest PA network in the Latin American and The Caribbean region.

For the Chilean case, in the middle of the nineteenth century, the rapid deforestation of south-central Chile, caused by land settlement and consequent agriculture and livestock activities, increased awareness about conservation [16]. Decree Law 18362 of 1984 created the national public system of PAs (*Sistema Nacional de Áreas Silvestres Protegidas del Estado*, SNASPE). The purpose of the law was to organize the scattered PAs around a unified conservation system with the common purpose of protecting Chilean natural resources. With the creation of SNASPE, the government tried to promote the definition and legalization of PAs boundaries and the assignment of specific management objectives for each unit in the system, so it can be considered as the true beginning of PAs in Chile [16].

Considering the different land uses represented in the Chilean system of PAs, almost 30% corresponds to forest. Chile has the largest temperate forest area in South America and more than half of the total area of temperate forests in the southern hemisphere [17]. This Chilean temperate forest has been classified as a biodiversity hotspot for conservation [18] and has also been included among the most threatened eco-regions in the world in the Global 200 initiative launched by WWF and the World Bank [19]. Most of the Chilean forests (including native and exotic forests) are distributed along the Coastal and the Andean Range of Chile from 35° to 56° totaling an area equal to 15.6 million ha [20].

In general for the case of Chile, the main driving force behind LULCC is the replacement of native forests by forest plantations and agricultural crops, a factor that contributes to soil loss, forest clearing, and the consequent loss of biodiversity [21]. In previous qualitative evaluations of SNASPE [16,21–24], authors have recognized that SNASPE geographic distribution and ecosystem representation is insufficient to achieve adequate levels of conservation and that the poor conservation status of native forests may be explained by the Chilean forest policies followed by the Law of Forest Development (Decree Law N° 701 of 1974). This law also aimed to promote forestation by small forest landowners to prevent soil loss, and to protect and restore soils. These policies have not provided economic incentives for the sustainable management and conservation of native forests, in contrast to the use of public funds to support the establishment of forest plantations.

At present, Chile has 14.1 million ha of PAs in its national public system which represents 18.7% of the Chilean territory. The system includes 31 national parks, 48 national reserves, and 15 natural monuments, with the first two categories encompassing 99.9% of the total area, and the system is administered by the Chilean Forest Service (*Corporación Nacional Forestal*, CONAF). Chilean protected areas rank second in Latin America and seventh worldwide in terms of percentage of national coverage [25] and according to the National Register of Protected Areas more than 90% of PAs included in SNASPE originate from government lands.

**Fig 1:**
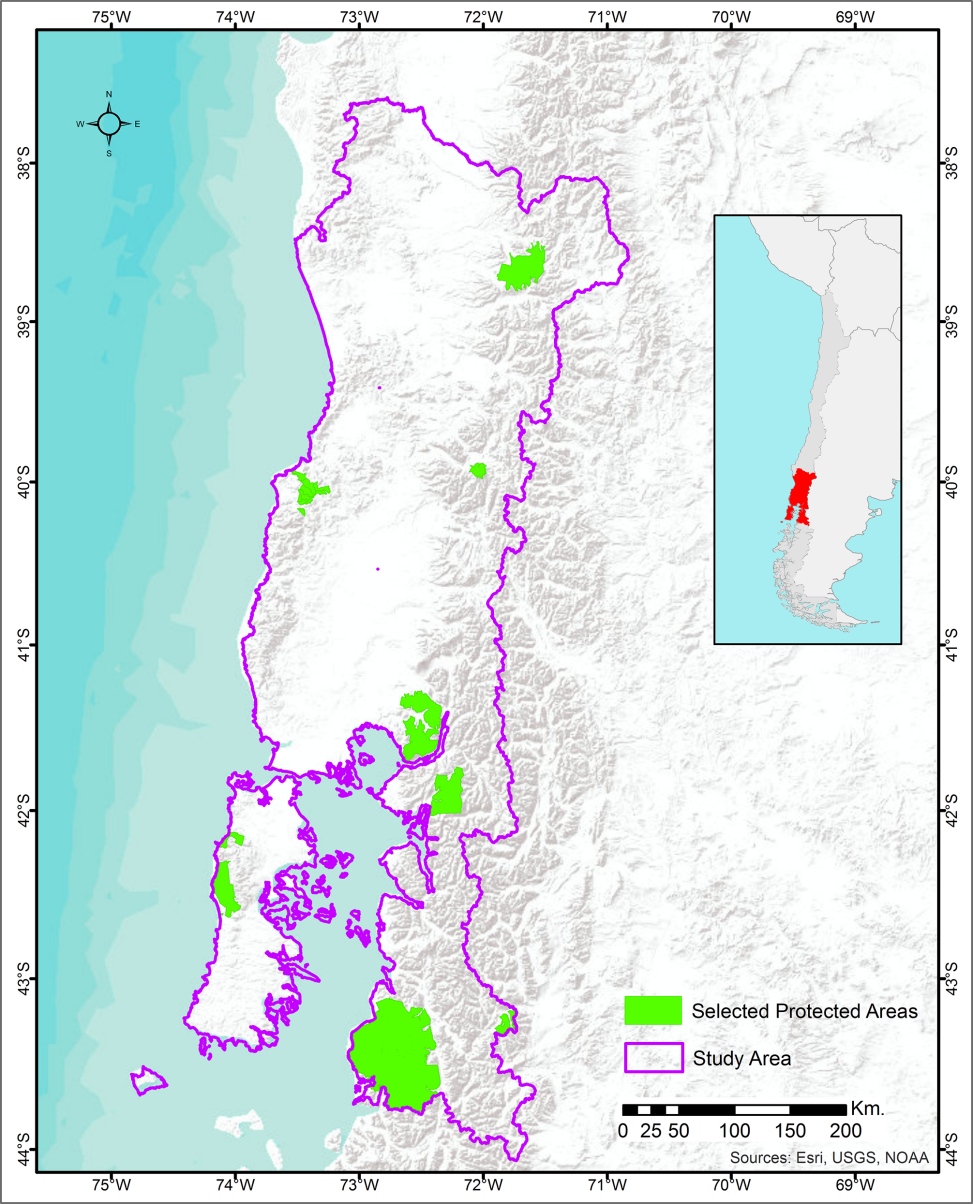
Map of the study area. Source: Base map from ESRI, USGS, NOAA (ArcGIS License 10.3).

In terms of criteria employed to establish PAs, the motivations have been diverse and have evolved to attain the current use of specific criteria and standards. However, at the beginning of the Chilean history of environmental protection, the criteria did not necessarily reflect conservation objectives, but rather they reflected a motivation to regulate wood extraction and commercialization, to protect public land without productive value (i.e. land with no capacity to sustain agriculture or cattle production), or to conserve landscape beauty [26]. Previous studies of the impact of Chilean PAs have not analyzed conservation effectiveness (i.e. what would have been the level of conservation had the PA not been established). Furthermore, there is no empirical evidence of the impact of the system which is an important knowledge gap that this paper aims to fill. Fig 1 shows the study area.

## Materials and Methods

### Data

The research estimated the causal impact on avoided deforestation of PAs established after the SNASPE creation in 1984. Until the 1970s, several government agencies were in charge of PAs creation and management. However, unified legislation on PAs was not available until 1984. With the creation of SNASPE, the government tried to promote the definition and legalization of PAs boundaries and the assignment of management objectives for each unit in the system, none of which previously had been clear for a large proportion of PAs [13]. For that reason, the SNASPE creation in 1984 can be considered the true beginning of PAs in Chile.

**Fig 2:**
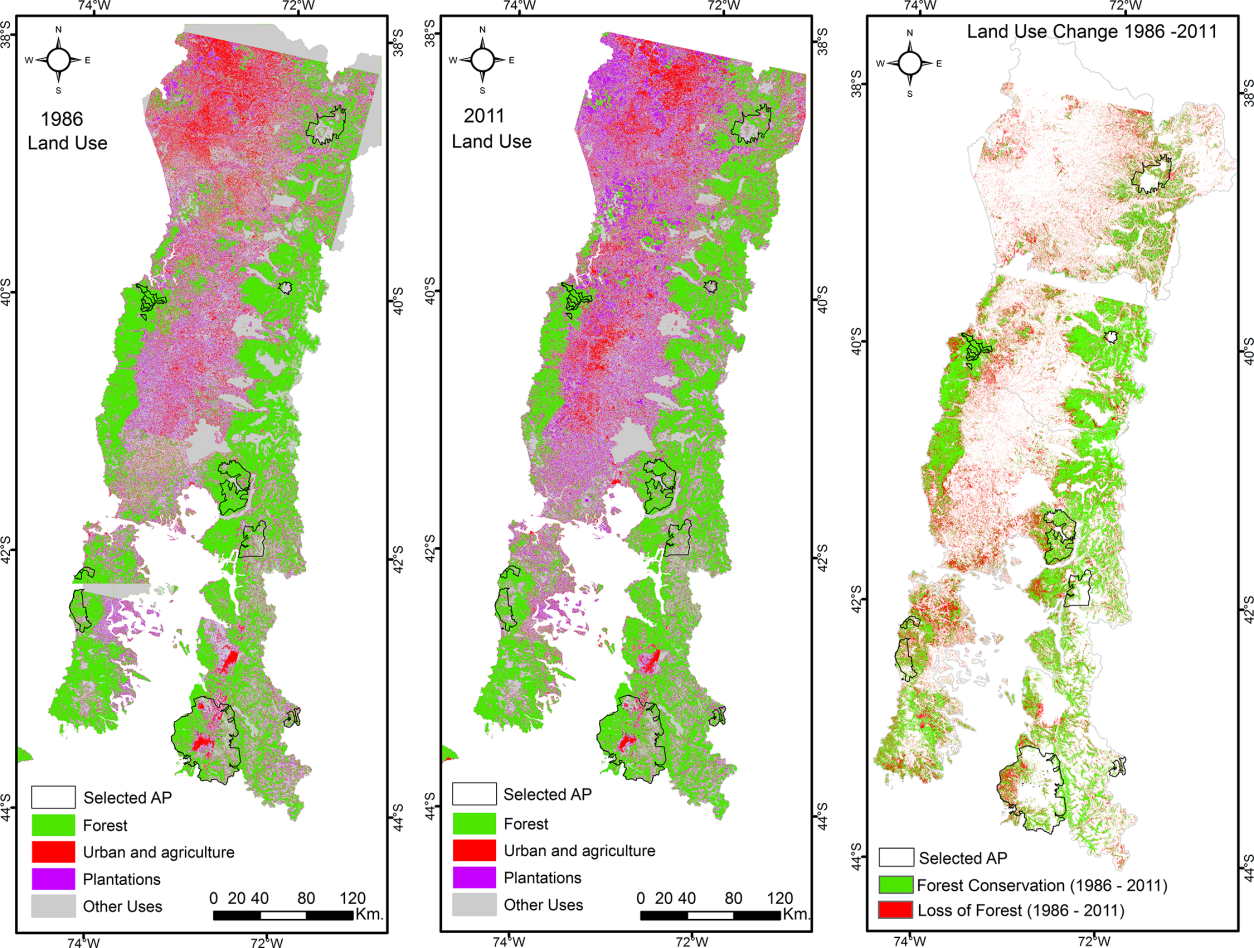
Land use and land cover changes between 1986 and 2011 in the study area. Source: Landscape Ecology Lab, Universidad de Concepcion, Chile. FONDECYT grant N°11110271.

The PAs located in the study area and selected for this study cover 561,920 ha. We used Geographic Information Systems (GIS) to build a geospatial data set of relevant biophysical and socioeconomic conditions. We first established the forest cover conditions using a mosaic of Landsat Multi-Spectral Scanner (MSS) satellite images between 1974 and 1976, and from 1986 and 2011 a mosaic of Landsat Thematic Mapper (TM) satellite images (Landscape Ecology Laboratory, *Universidad de Concepción*, Chile) (see Fig 2). MSS images consist of four spectral bands with 60 meter spatial resolution and TM images consist of seven spectral bands with a spatial resolution of 30-meter pixels. The MSS pixels were resampled to make them comparable to TM pixels. A random sample of 2,549 and 36,417 points (pixels) was obtained to characterize protected and unprotected land respectively. These treated and control points were selected to well represent the study area and to include approximately one pixel per 1 km2 of land within the study area. After removing points that were not usable because of the land use change obtained from the satellite images classification (e.g. a forested point in 1986 without data in the 2011 satellite image), the final dataset comprised 1,978 treated points covering all protected areas included in the analysis and 23,181 control points. To determine if a land pixel is considered protected for the analyses, a layer containing all PAs was overlaid with a general map of the study area. The sampling excluded indigenous land and private PAs because they are subject to different legal and land use regimes. For the purpose of this analysis, a private PA is a piece of land of any size that: (i) is managed with the purpose of conserving biodiversity; (ii) is protected with or without formal recognition from the government; and (iii) is managed directly or indirectly by individuals, communities, corporations or non-governmental organizations.

To check the accuracy of the random sampling process, we confirmed that there were no significant differences between our sample of land pixels and the entire land area shown in Fig 1 in terms of important characteristics (i.e., protected status, type of protection, proportion under each land capacity and land suitability classes).

Deforestation between 1986 and 2011 was calculated based on a forest cover variable, defined as the presence or absence of forest at the pixel level (i.e., a binary variable indicating if a pixel is either forested or deforested in each year). We took land that was forested in 1986 and compared deforestation in protected and unprotected forests. As a result, the outcome variable measured the change in forest as the difference between the change in forest cover on protected pixels (Y = 1 if conserved) and the change in forest cover on matched unprotected pixels in the same period (1986–2011). Thus, a positive sign indicates that protection resulted in avoided deforestation. Table 1 shows, through a simple comparison between protected and unprotected areas, a higher probability of conservation on unprotected land for the period 1975–1986, and a higher probability of conservation on protected land for the period 1986–2011. These results do not allow for a conclusion about PAs’ impacts in terms of avoided deforestation, but they show statistically significant differences in terms of the outcome variable between protected and unprotected areas in the study region.

**Table 1:** Description and summary statistics for avoided deforestation.

Variable Description	Mean Protected land	Mean Unprotected land	t-stat	Norm Diff^*b*^
Deforestation between 1975–1986 ^*a*^	0.757 (0.430)	0.827 (0.378)	2.589	-0.172
Deforestation between 1986–2011 ^*a*^	0.892 (0.310)	0.723 (0.447)	-15.899	0.544

^*a*^ These outcomes show the difference between the change in forest cover on protected plots (Y = 1 if not deforested) and the change in forest cover on matched unprotected plots in the same period. Thus, a positive sign indicates that protection resulted in higher probability of conservation or avoided deforestation.

^***b***^ Normalized difference = $\frac{{\bar{X}}_{T}-{\bar{X}}_{C}}{\sqrt{\frac{S_{T}^{2}+S_{C}^{2}}{2}}}$ where T = protected and C = unprotected [27].

To control for differences among protected and unprotected areas in terms of characteristics that affect both deforestation and protection decisions, the forest cover data was combined with spatially explicit data on covariates believed to affect both PA location and LULCC. The biophysical, geographical and socioeconomic characterization of Chilean PAs is oriented to reveal the drivers of LULCC and conservation status in Chile when compared with non-protected areas. In the scientific literature, the main drivers of PAs establishment are related to land use [9,10], soil characteristics [28,29] and transportation costs [9,28–31]. Other drivers may include ecological characteristics like slope and distance to rivers [9,29,31]. We also draw on previous impact evaluation of PAs [9,10,13,32]. For the purpose of this paper, variables describing terrain, climate, and remoteness were used to compare protected land with unprotected land as shown in Table 2, and Table 3 presents the summary statistics for confounders used in our analysis.

**Table 2:** Definitions of variables and data sources for drivers of the establishment of PAs.

Variable	Definition	Data source
**Socioeconomic drivers of PAs establishment**		
Distance to river	Euclidean linear distance to the closest river	Ministry of the Interior (2002), scale: 1:20,000
Distance to closest city	Euclidean linear distance to the border of the closest urban city	Ministry of the Interior (2002), scale: 1:20,000
Distance to road	Euclidean linear distance to the closest national highway	Adapted from the Ministry of Public Works (2012)*
**Biophysical drivers of PAs establishment**		
Altitude	Mean value of sampled pints using a GIS layer with terrain elevation using meters at the sea level (MASL) as measurement unit with a spatial resolution of 30 and 90m	Terrain Elevation Model (TEM) years 2008 and 2001, International Centre for Tropical Agriculture (CIAT)
Slope	Mean value of sampled points using a GIS layer with terrain elevation using an angle of inclination to the horizontal (degrees) as measurement unit with a spatial resolution of 30 and 90m	Terrain Elevation Model (TEM) years 2008 and 2001, International Centre for Tropical Agriculture (CIAT)
Precipitation	Annual precipitation (mm)	Universidad de La Frontera, Temuco, Chile (2004), scale: 1:250,000
High soil erodibility	Proportion of sampled points with very high and high soil erodibility	National Commission of the Environment, scale: 1:250,000
Medium soil erodibility	Proportion of sampled points with very moderate soil erodibility	National Commission of the Environment, scale: 1:250,000
Low soil erodibility	Proportion of sampled points with very low and very low soil erodibility	National Commission of the Environment, scale: 1:250,000

* The distance to roads was calculated from an adaptation of a data set from the Ministry of Public Works (2012). The adaptation process involved the use of a road cadastral map from 1969. This map allowed us to identify the road network that existed in Chile in 1969 which was the road network used as a covariate during the matching process.

**Table 3:** Covariate Balance.

Variable	Sample^*a*^	Mean Value Protected Area	Mean Value Unprotected Area^*b*^	Diff Mean Value	Avg. Raw eQQ Diff^*c*^	Mean eCDF Diff^*d*^	Norm diff^*e*^
Distance to river (km)	Unmatched	2.54	2.25	0.29	0.29	0.03	0.13
	Matched	2.14	2.10	0.04	0.06	0.01	0.02
Distance to closest city (km)	Unmatched	37.63	22.88	14.75	14.83	0.22	0.81
	Matched	30.06	29.52	0.54	0.89	0.02	0.03
Distance to road (km)	Unmatched	32.48	40.39	-7.91	8.33	0.09	-0.34
	Matched	33.39	34.15	-0.76	1.28	0.02	-0.04
Altitude (masl)	Unmatched	645.93	528.70	117.23	117.88	0.07	0.29
	Matched	670.60	657.65	12.95	14.111	0.01	0.03
Slope (°)	Unmatched	19.54	15.16	4.38	4.37	0.09	0.37
	Matched	18.56	18.45	0.11	0.47	0.01	0.01
Precipitation (mm)	Unmatched	2190.00	1942.10	247.90	253.97	0.22	0.86
	Matched	2147.10	2141.30	-0.20	22.07	0.02	0.02
Temperature (°C)	Unmatched	3.88	-10.91	14.79	21.39	0.14	0.05
	Matched	4.06	4.32	-0.26	0.27	0.05	-0.18
High soil erodibility^*f*^	Unmatched	0.92	0.46	0.45	0.45	0.23	1.13
	Matched	0.91	0.91	0.00	0.00	0.00	0.00
Low soil erodibility^*f*^	Unmatched	0.08	0.43	-0.35	0.35	0.17	-0.87
	Matched	0.09	0.09	0.00	0.00	0.00	0.00

^*a*^ N treated = 1978; N available controls = 23181.

^*b*^ Weighted means for matched controls.

^***c***^ Mean (for categorical covariate) or median (for continuous covariate) difference in the empirical quantile-quantile plot of treatment and control groups on the scale in which the covariate is measured.

^*d*^ Mean eCDF = mean differences in empirical cumulative distribution function.

^*e*^ Normalized difference = $\frac{{\bar{X}}_{T}-{\bar{X}}_{C}}{\sqrt{\frac{S_{T}^{2}+S_{C}^{2}}{2}}}$ where T = protected and C = unprotected [27].

^*f*^ According to FAO, the erodibility of a soil as a material with a greater or lesser degree of coherence is defined by its resistance to two energy sources: the impact of raindrops on the soil surface, and the shearing action of runoff between clods in grooves or rills (see http://www.fao.org/docrep/t1765e/t1765e0f.htm).

### Empirical strategy

We wanted to estimate the difference between the expected potential change in forest cover on protected land and the counterfactual expected potential change in forest cover (i.e., what would have happened had the PA not been created) on unprotected areas. To ensure an appropriate causal analysis, we used four strategies: (i) comparison of means, (ii) conducting statistical matching, (iii) adjusting for remaining bias post-matching, and (iv) testing for unobservables that may bias causal estimates.

Following [9] and [33], and given that protection is influenced by observable characteristics which also affect deforestation, we used matching methods to estimate avoided deforestation. Matching methods are becoming increasingly applied in the impact evaluation literature as a method to establish cause–effect relationships with nonexperimental data [34]. Matching works by comparing conservation outcomes on protected and unprotected forest plots that are very similar in terms of the observed baseline characteristics. The goal of matching is to make the distribution of characteristics of protected and unprotected plots similar which is called covariate balancing. Matching can be viewed as a method to make the protected and unprotected covariate distributions look similar by reweighting the sample observations (e.g., unprotected plots that are poor matches receive a weight of zero). Thus, matching mimics random assignment through the ex post construction of a control group [9].

We used a simple difference-in-differences (DID) estimator (called the Before-After-Control-Impact estimator in the ecology literature), which can control for time-invariant unobservable characteristics. When estimating the causal impact of PAs with the simple DID estimator, the key identification assumption is that the expected trend in forest cover of the unprotected land is equal to the expected trend in forest cover of the PAs in the absence of the program. To make this assumption more plausible, we first characterized all control unprotected land in our sample based on the selection criteria for PAs and then selected land based both on these criteria and the rule that protected and unprotected areas should be forested at the baseline, thus making treated and control areas more similar at baseline (a form of “pre-matching”). Deforestation trends before the creation of SNASPE was also compared between protected and unprotected land using the 1974–1976 classified satellite images described in the previous section.

Based on an assessment of covariate balance quality across a variety of matching methods [35,36], we chose one-to-one, nearest-neighbor covariate matching with replacement using a generalized version of the Mahalanobis distance metric and genetic matching algorithm that maximizes covariate balance. Matching was done in R. Bootstrapped standard errors are invalid with non-smooth, nearest-neighbor matching with replacement [37], and thus we used Abadie and Imbens’ variance formula to conduct a t-test of the mean difference-in-differences [38].

Matching estimators of PAs’ impacts may still be biased due to discrepancies between the covariates of the matched protected pixels and their unprotected matches. We reduced this bias by using regression methods for the matched data. The use of a post-matching bias correction procedure asymptotically removes the conditional bias in finite samples [38], although this use of regression is different from its use in the full sample. Here the covariate distributions are likely to be similar in the matched sample, and so regression is not used to extrapolate out of sample. This strategy of post-matching regression adjustment typically generates treatment effects estimates that are more accurate and more robust to misspecification than parametric regression alone [35,39].

This empirical design compared PAs with observationally similar unprotected lands (after removing non-comparable areas, such as indigenous reserves or private PAs), but without distinguishing between private and government lands as comparable control unprotected areas. This empirical strategy ignores that traditional PA systems are usually based on converted, long-held government lands which perhaps have different land use regimes relative to private lands. In order to test if estimates of the impacts of PAs vary when using only public land as control unprotected areas, the causal analysis described above was repeated using only public lands to build a control group and to compare means, to conduct statistical matching, and to adjust for remaining bias post-matching.

Despite the efforts to control for observable sources of bias, protection and changes in LULCC may show correlation in the absence of an impact of protection because of failure to match on a relevant but unobserved covariate. In an analysis of this kind, the main concern is that PAs may be unobservably less likely to be deforested than their matched unprotected areas [9]. Sensitivity analysis examines the degree to which uncertainty about hidden biases in the assignment of protection could alter the conclusions of this study. We used the sensitivity test proposed by [40] and based on the Wilcoxon statistical test that assumes that each observation unit has a fixed value of one unobserved covariate (or a compound of unobserved covariates). The unobserved covariate not only affects protection assignment, but also determines whether deforestation is more likely for the protected cells or their matched controls. Thus, this sensitivity test is conservative [9]. Matched forested pixels differ in their odds of being protected by a factor of Г as a result of this unobserved covariate (Г = 1 in the absence of hidden bias).The higher the gamma (Г) level range at which the inference of the estimated effect of PAs on deforestation does not change, the more confidence there will be that the conclusion regarding the estimation of causal effect is not affected by an unobserved difference.

## Results

Table 4 presents the DID matching estimates of avoided deforestation as a proportion of forest conserved. Estimates based on matching methods were compared with estimates based on more conventional methods used in conservation science literature. In the second column are estimates for PAs established after SNASPE creation (1984). Decree Law 18,362 of 1984 created SNASPE, however, program implementation took time after that date. For the purpose of this study, 1986 data provide the pre-SNASPE information necessary to construct baseline forest cover scenarios for both protected and unprotected areas. The first row presents the avoided deforestation estimates generated by conventional methods used in the conservation science literature where deforestation on protected plots is compared with deforestation on unprotected areas without controlling for other covariates. It implies that 16.9% of protected plots would have been deforested by 2011 in the absence of protection.

**Table 4:** Estimated avoided deforestation as a proportion of forest protected.

	Protected after 1986 (control: never protected and forested in 1986)	Protected after 1986 (control: public land never protected and forested in 1986)
**Conventional conservation science approach**		
Difference in means^*a*^	0.169**	0.011
[N protected pixels]	[1978]	[1978]
{N available controls}	{23181}	{339}
**Sample selected by covariate matching**		
Difference in means^*b*^	0.047* (0.021)	0.023 (0.081)
[N matched controls]	[1978]	[1978]
**Sample selected by covariate matching with calipers**^***c***^		
Difference in means^*b*^	0.048**	-0.008 (0.029)
[N outside calipers]	[714]	[1018]
{N matched controls with calipers}	{1264}	{960}
Marginal effect from multivariate regression^*d*^	0.047** (0.013)	-0.004 (0.013)

^*a*^ Statistical significant difference in means evaluated with a Chi-squared test between treated and control sub-samples.

^*b*^ Standard errors for matching estimates using Abadie-Imbens standard error formula [38]

^*c*^ Calipers restrict matches to units within 0.5 standard deviations of each covariate.

^*d*^ OLS regression on avoided deforestation, with covariates including all variables used in covariate matching.

* Significance at 5%.

** Significance at 1%.

**Fig 3:**
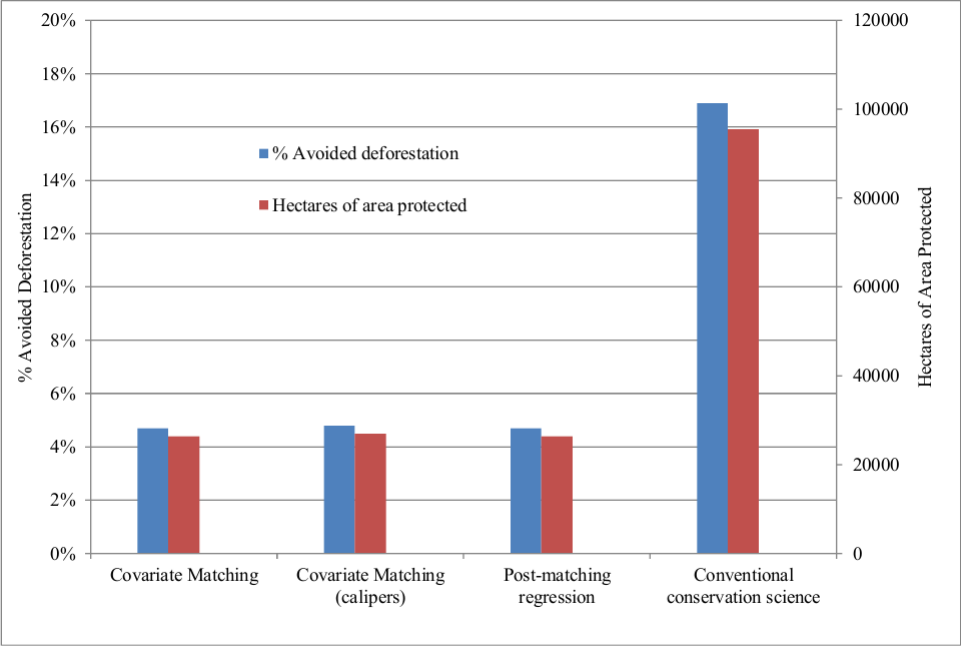
Avoided deforestation estimates 1986–2011. The estimates of avoided deforestation estimates are based on the different matching methods in Table 4 including the estimates from the conventional method used in the conservation science literature where deforestation on protected plots is compared with deforestation on unprotected areas without controlling for other covariates.

Nevertheless, as seen in Table 3, the protected and unprotected areas differ on key covariates that could affect not only protection status but also changes in forest cover. In terms of covariates associated with protection status and deforestation, Table 3 shows that there are statistically significant differences between protected and unprotected land across all the socioeconomic and biophysical drivers associated with protection status. In general, PAs are farther from cities and road networks, and are located on steeper and higher land. Similarly, PAs also tend to be placed on land less suitable for agriculture and on soil with higher erodibility. Thus one might worry that despite our pre-matching efforts and the similar baseline forest cover before protection, the mean avoided deforestation among protected land from 1986 to 2011 in the absence of protection, may not be well represented by the mean change in forest cover for unprotected lands during the same period. In fact, avoided deforestation estimates from matching approaches were much smaller. Covariate matching estimates, with and without calipers, implied that only 4.7% and 4.8% of protected plots would have been deforested by 2011 in the absence of protection respectively. Matching with calipers was used to further address concerns about potential bias where calipers define a tolerance level for judging the quality of the matches: if available controls are not good matches for a treated unit (i.e., there is no match within the caliper), the unit is eliminated from the sample. In our study, we view calipers as a robustness check. We define the caliper as 0.5 standard deviations of each matching covariate. Matching estimates could still be because even after matching, there are statistically significant differences in the observable baseline characteristics of PAs and matched controls. Table 3 shows several metrics of covariate balance before and after matching for the sample. The fifth and sixth columns of Table 3 present two measures of the differences in the covariate distributions between protected and unprotected areas, namely, the difference in means and the average distance between the two empirical quantile functions (values greater than 0 indicate deviations between the groups in some part of the empirical distribution). If matching is effective, these measures should move towards zero [35], which is what we observe. The last column of Table 3 also shows normalized differences. By using a post-matching bias correction procedure that asymptotically removes the conditional bias in finite samples, the last row of Table 4 shows that 4.7% of protected plots would have been deforested by 2011 in the absence of protection, which is not much different from the estimates with matching without the bias correction procedure. To put Table 4‘s estimates into perspective, consider that 561,920 ha of forest were protected between 1984 and 2011. Thus, conventional methods imply 95,526 ha of avoided deforestation. In contrast, the matching methods imply 26,410–26,972 ha of avoided deforestation (see Fig 3).

The third column of Table 4 presents DID matching estimates using public land never protected and forested in 1986 as control. Neither estimates from conventional conservation science approaches nor from samples selected by matching show significant estimates of PAs’ impact on circumvented deforestation between 1986 and 2011. Table 5 shows the covariate balance for this alternative causal analysis.

**Table 5:** Covariate Balance for public land.

Variable	Sample^*a*^	Mean Value Protected Area	Mean Value Unprotected Area^*b*^	Diff Mean Value	Avg. Raw eQQ Diff^*c*^	Mean eCDF Diff^*d*^	Norm diff^*e*^
Distance to river (km)	Unmatched	2.54	2.56	-0.02	0.25	0.02	-0.13
	Matched	2.54	2.30	0.24	0.29	0.03	0.10
Distance to closest city (km)	Unmatched	37.63	64.05	-26.42	26.41	0.29	-0.68
	Matched	37.63	38.26	-0.63	3.34	0.05	-0.01
Distance to road (km)	Unmatched	32.48	48.61	-16.13	18.46	0.11	-0.58
	Matched	32.48	49.24	-16.76	16.85	0.21	-0.20
Altitude (masl)	Unmatched	645.93	477.29	168.64	176.92	0.12	-0.02
	Matched	645.93	556.98	88.95	106.19	0.07	0.07
Slope (°)	Unmatched	19.54	18.90	0.64	1.16	0.03	-0.07
	Matched	19.54	19.52	0.02	1.23	0.03	0.01
Precipitation (mm)	Unmatched	2190.00	1811.00	379.00	402.91	0.30	1.27
	Matched	2190.00	2195.80	-5.80	40.80	0.04	0.26
Temperature (°C)	Unmatched	3.88	3.40	0.48	0.87	0.14	0.50
	Matched	3.88	3.07	0.81	0.36	0.05	0.17
High soil erodibility^*f*^	Unmatched	0.92	1.00	-0.08	0.08	0.04	-0.27
	Matched	0.92	1.00	-0.08	0.08	0.04	-0.27
Low soil erodibility^*f*^	Unmatched	0.08	0.00	0.08	0.08	0.04	0.27
	Matched	0.08	0.00	0.08	0.08	0.04	0.27

^*a*^ N treated = 1978; N available controls = 23181.

^*b*^ Weighted means for matched controls.

^***c***^ Mean (for categorical covariate) or median (for continuous covariate) difference in the empirical quantile-quantile plot of treatment and control groups on the scale in which the covariate is measured.

^*d*^ Mean eCDF = mean differences in empirical cumulative distribution function.

^*e*^ Normalized difference = $\frac{{\bar{X}}_{T}-{\bar{X}}_{C}}{\sqrt{\frac{S_{T}^{2}+S_{C}^{2}}{2}}}$ where T = protected and C = unprotected [27].

^*f*^ According to FAO, the erodibility of a soil as a material with a greater or lesser degree of coherence is defined by its resistance to two energy sources: the impact of raindrops on the soil surface, and the shearing action of runoff between clods in grooves or rills (see http://www.fao.org/docrep/t1765e/t1765e0f.htm).

Table 6 presents results from sensitivity tests that assess the degree to which potential unobservable heterogeneity makes us uncertain about the inferences drawn in Table 4. A sensitivity analysis like this asks: How would inferences about the effects of PAs be altered by hidden biases of various magnitudes? How large would these differences have to be to alter the conclusions of the study? Table 6 presents the analysis for the estimates from matching with calipers. The second column in Table 6 indicates that the estimate of 4.8% avoided deforestation remains significantly difference from zero even in the presence of moderate unobserved biases. To attribute the avoided deforestation to an unobserved covariate rather that to an effect of PAs, that unobserved covariate would need to produce more than a 1.35 increase in the odds ratio of protection to differ between protected and unprotected cells, the 95% confidence interval would still exclude zero.

**Table 6:** Sensitivity test of hidden biases measured by critical p-values.

Γ	Protected in 2011 (never protected and forest in 1986 control)
1.00	0.0000
1.05	0.0000
1.10	0.0001
1.15	0.0005
1.20	0.0023
1.25	0.0080
1.30	0.0226
1.35	0.0536
1.40	0.1087
1.45	0.1921
1.50	0.3019
1.55	0.4287
1.60	0.5592
1.65	0.6799

The sensitivity test presented in Table 6 is free of parametric assumptions and provide an easily interpretable measure of the way in which an unobservable covariate may bias a result. In this case, the assumed unobserved covariate is a strong confounder: one that not only affects PAs selection but also determines whether deforestation is more likely for the protected units or their matched unprotected areas. Rosenbaum bounds are in this sense a ‘‘worst-case” scenario [41]. Nonetheless, they convey important information about the level of uncertainty contained in matching estimators by showing just how large the influence of a confounding variable must be to undermine the conclusions of a matching analysis

## Discussion and Conclusions

Conservation practitioners and policymakers need credible information on how PA policies affect ecosystems. The primary question PA administrators should ask is whether the conservation scheme adds a difference in conservation between the with-PAs scenario and the without-PAs baseline [15]. However, the level of additionality that a PAs network can provide also depends on where protection is being placed. Most previous evaluations on PAs’ effectiveness have relied on indirect estimates based on comparisons between protected and unprotected areas. However, such methods could easily be biased when protection is not randomly assigned, but rather is determined by characteristics that also affect LULCC (e.g., land productivity, accessibility). Our results confirmed that conventional scientific approaches claim land cover impacts for protection which are actually due to PA networks’ landscape characteristics.

For Chile, the state of conservation of its natural resources is a topic of growing concern among the general public as well as national and international conservation organizations. Previous analysis of Chilean PAs have focused only on ecosystem representation, coverage of biodiversity hot-spots, budgets, and boundary issues. However, conservation additionality of PAs has not been mentioned in any previous studies. For this case, we show that selection bias exists in assigning protection, which might then result in fewer additional conservation benefits to one of the oldest systems of PAs in Latin America. Chilean PAs are farther from cities and road networks, and are located on steeper and higher land. They tend to be placed on land less suitable for agriculture and on soil with higher erodibility.

In terms of methodological lessons, results show the importance of the comparison group (i.e., selection of appropriate unprotected land to be used as controls to match PAs). In that sense, finding causal impacts of PAs in terms of forest conservation may well depend on the selection of the appropriate control group. Our analysis illustrates how substantial improvements can be made to estimates concerning the impact of PAs. However, in estimating forest conservation effectiveness, the selection of appropriate unprotected land to compare with the outcome observed in PAs is very important. By comparing PAs with observationally similar unprotected lands (after removing non-comparable areas, such as indigenous reserves or private PAs) as done in previous research, one ignores that traditional PA systems are usually based on converted, long-held government lands which perhaps have different land use regimes relative to private lands. The causal analysis, using only public lands to build a control group, showed no difference between the conservation outcome obtained with PAs and the conservation outcome that would have happened had the PAs not been established. In the Chilean context, regulatory regimes are similar between PAs and public lands. Public lands similar to PAs are well managed and so converting these lands to PAs does not necessarily provide additional conservation benefits. These results raise important questions, such as the relative costs of different types of public land management, and whether there are any particular types of public lands where creating PAs would have a greater impact in terms of avoided deforestation. These results also suggest that conversion of private land to PAs may offer more avenues in terms of additional conservation benefits when compared to the conservation scenario including only public lands. If forests in public lands are almost as well protected as they are in PAs, as our analysis shows, the Chilean government would do well strategically to commit to keeping natural forests on public lands alongside the PA network. However, the inclusion of private land into the system of PAs may offer a higher level of supplementary conservation benefits. Therefore, the promotion of incentives to include private PAs could be very important, particularly considering that the expropriation of private land for conservation purposes is less feasible given the financial constraints of a system with very limited resources to invest in conservation. In fact, a new Biodiversity and Protected Areas Law is currently under discussion in the Chilean congress. One of the new objectives of this law refers to the inclusion of a private network of PAs into a new integrated national system of PAs.

In some countries, PAs can be assigned without adequate financial resources, or without the infrastructure and networking needed to substitute consumable resources and protect the area from development or misuse. Moreover, PAs are biased towards places where they can least prevent land conversion. Often it may be financially and politically expedient to protect public land with low financial value and to avoid places with conflictive land use alternatives when considering conservation objectives [4]. In the Chilean context, PAs established in the study area came from public land set aside for conservation purposes. Although the SNASPE law includes private land expropriation for conservation objectives, the history of PAs in Chile does not show evidence of this kind of practice. As a result, the current system of PAs originates almost exclusively from public land. Therefore, counterfactual scenarios obtained from forest cover changes on matched unprotected private lands may not reflect the same results one may obtain if these counterfactual scenarios are constructed using unprotected public lands as potential control unprotected areas.

Given that not all PAs are established to avoid deforestation, results also admit the potential for positive outcomes. Therefore, impact evaluations of PAs should also work toward developing additional measures for program outcomes including forest degradation (not just deforestation), biodiversity, wildlife, primary forests (not forests in general), among others.

## Supporting Information

S1 File Data. This file contains all data necessary to replicate the underlying findings reported in this paper.
(TXT)
